# Detection and Severity Assessment of Peripheral Occlusive Artery Disease via Deep Learning Analysis of Arterial Pulse Waveforms: Proof-of-Concept and Potential Challenges

**DOI:** 10.3389/fbioe.2020.00720

**Published:** 2020-06-30

**Authors:** Sooho Kim, Jin-Oh Hahn, Byeng Dong Youn

**Affiliations:** ^1^Department of Mechanical and Aerospace Engineering, Seoul National University, Seoul, South Korea; ^2^Department of Mechanical Engineering, University of Maryland, College Park, MD, United States; ^3^OnePredict, Inc., Seoul, South Korea

**Keywords:** peripheral artery disease, cardiovascular disease, deep learning, machine learning, pulse wave analysis, arterial hemodynamics, ankle-brachial index, convolutional neural network

## Abstract

Toward the ultimate goal of affordable and non-invasive screening of peripheral occlusive artery disease (PAD), the objective of this work is to investigate the potential of deep learning-based arterial pulse waveform analysis in detecting and assessing the severity of PAD. Using an established transmission line model of arterial hemodynamics, a large number of virtual patients associated with PAD of a wide range of severity and the corresponding arterial pulse waveform data were created. A deep convolutional neural network capable of detecting and assessing the severity of PAD based on the analysis of brachial and ankle arterial pulse waveforms was constructed, evaluated for efficacy, and compared with the state-of-the-art ankle-brachial index (ABI) using the virtual patients. The results suggested that deep learning may diagnose PAD more accurately and robustly than ABI. In sum, this work demonstrates the initial proof-of-concept of deep learning-based arterial pulse waveform analysis for affordable and convenient PAD screening as well as presents challenges that must be addressed for real-world clinical applications.

## Introduction

Peripheral artery occlusive disease (PAD) is a highly prevalent vascular disease associated with high morbidity and mortality risks. It was estimated that >8 million and >200 million people were suffering from PAD in the United States (in 2000) (Allison et al., 2007) and globally (in 2010) (Fowkes et al., 2013), and the number of PAD patients is projected to sharply increase with societal aging. It makes a significant adverse impact on morbidity and quality of life, and also carries significant mortality implications as a powerful predictor of coronary artery disease and cerebrovascular disease (Golomb et al., 2006). Nonetheless, PAD is underdiagnosed with low primary care awareness (Hirsch et al., 2001).

In clinical practice today, PAD diagnosis necessitates angiography techniques (Guthaner et al., 1983; Romano et al., 2004; Cavallo et al., 2019). These techniques are not ideally suited to affordable and convenient PAD detection and severity assessment. The current gold standard is the digital subtraction angiography, which is an invasive technique. Other non-invasive imaging-based angiography techniques including the computed tomography angiography and magnetic resonance angiography require X-ray radiation and expensive equipment not appropriate for affordable settings. The ankle-brachial index (ABI) is a relatively low-cost technique and is widely used for PAD screening. However, it is often criticized for its limited accuracy and robustness in diagnosing PAD (Nelson et al., 2012).

Machine learning (ML) is increasingly exploited in cardiovascular disease (CVD) detection and prognosis. In particular, ML has exhibited promising efficacy in heart disease detection and prediction (Dogan et al., 2018; Abdar et al., 2019; Vallée et al., 2019) as well as CVD risk and CV death prognosis (Ambale-Venkatesh et al., 2017; Steele et al., 2018; Alaa et al., 2019). Recent reports increasingly exploit deep learning (DL) to capitalize on its ability to automatically select characteristic features, especially in conjunction with medical imaging techniques (Abdolmanafi et al., 2018; Poplin et al., 2018; Zhang et al., 2019). In contrast to the large body of existing work on ML-based CVD detection and CV mortality prediction, relatively small number of work on ML applications to PAD is available, including detection and mortality prognosis using electronic health record as well as genomic and imaging data (Ross et al., 2016; Arruda-Olson et al., 2018).

The analysis of arterial pulse waveforms [called hereafter the pulse waveform analysis (PWA)] may play a complementary role to ML in PAD diagnosis. In fact, our prior work shows that model-based PWA has the potential to estimate CV risk predictors (Ghasemi et al., 2018) and diagnose CVD (Ebrahimi Nejad et al., 2017) using diametric arterial pulses. A recent work illustrated the theoretical feasibility of PAD diagnosis (including detection, localization, and severity assessment) using a hybrid model- and ML-based analysis of central aortic and peripheral arterial pulses (Xiao et al., 2016a). A practical advantage of PWA is that it may be relevant to affordable PAD screening and diagnosis with convenient arterial pulse measurements at the extremity locations (e.g., arm and ankle).

Despite the complementary value of DL and PWA in advancing the diagnosis of PAD (and even other CVDs), the fusion of DL and PWA for PAD diagnosis has never been pursued to the best of our knowledge. In fact, the state-of-the-art of DL-based PWA appears to be limited to rudimentary classification of CV health state (e.g., hypertension, atherosclerosis, and diabetes mellitus) (Li et al., 2019). Hence, DL-PWA fusion is a novel conceptual idea worthy of pursuit in the context of CVD diagnosis (including PAD).

Toward the long-term goal of affordable and non-invasive PAD screening and diagnosis, the objective of this work is to investigate the potential of DL-based arterial PWA in detecting and assessing the severity of PAD. Using an established transmission line (TL) model of arterial hemodynamics, a large number of virtual patients associated with PAD of a wide range of severity and the corresponding arterial pulse waveform data were created. A deep convolutional neural network (CNN) capable of detecting and assessing the severity of PAD based on the analysis of brachial and ankle arterial pulse waveforms was constructed, evaluated for efficacy, and compared with the state-of-the-art ABI using the virtual patients.

This paper is organized as follows. Section “Materials and Methods” presents a multi-branch TL model of arterial hemodynamics used in this work, creation of virtual PAD patients together with the corresponding arterial pulse waveforms to investigate DL-based PWA for PAD diagnosis, a DL-based PWA approach based on the CNN for PAD detection and severity assessment, and data analysis methods to evaluate the efficacy of the DL-based PWA approach. Section “Results” presents results, which are discussed in section “Discussion.” Section “Conclusion” concludes this work with future directions.

## Materials and Methods

### Transmission Line Model of Arterial Hemodynamics

**FIGURE 1:**
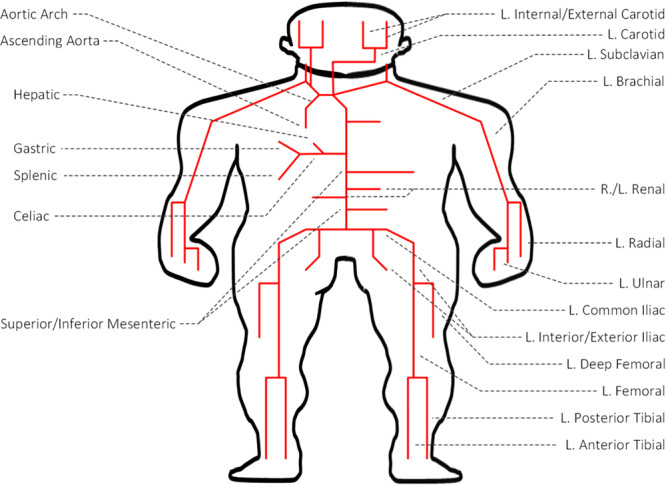
Transmission line (TL) model of arterial hemodynamics consisting of 55 TLs, each of which represents an arterial segment characterized by segment-specific viscous, elastic, and inertial properties.

We used a multi-branch TL model of arterial hemodynamics developed in a prior work (Figure 1; He et al., 2012). In brief, the model is composed of 55 TLs, each of which represents an arterial segment characterized by segment-specific viscous, elastic, and inertial properties. In each TL, the propagation of arterial blood pressure (BP) and flow (BF) waves is dictated by the propagation and reflection constants as well as the arterial length:

(1) $$\begin{array}{c} {\text{p}}_{\text{O}}={\text{p}}_{\text{I}}\,\left(1+\text{Γ}\right)\,/\,\left({\text{e}}^{\gamma \,l}+\Gamma \,e^{-\gamma \,l}\right)\\ {\text{q}}_{\text{O}}={\text{q}}_{\text{I}}\,\left(1-\text{Γ}\right)\,/\,\left({\text{e}}^{\gamma \,l}-\Gamma \,e^{-\gamma \,l}\right) \end{array}$$

where p_I_ and p_O_ are BP waves at the inlet and outlet of the artery, q_I_ and q_O_ are BF waves at the inlet and outlet of the artery, γ is the propagation constant, Γ is the reflection constant, and l is the arterial length. BP and BF waves at the inlet of the artery are related by the input impedance of the arterial segment:

(2) $${\text{p}}_{\text{I}}={\text{q}}_{\text{I}}\,{\text{Z}}_{\text{I}}={\text{q}}_{\text{I}}\,{\text{Z}}_{\text{C}}\,\left({\text{e}}^{\gamma \,l}+\Gamma \,e^{-\gamma \,l}\right)\,/\,\left({\text{e}}^{\gamma \,l}-\Gamma \,e^{-\gamma \,l}\right)$$

where Z_I_ and Z_C_ are the input impedance and characteristic impedance of the artery, respectively. If an arterial segment is terminated by a bifurcation, its load impedance is given by the parallel connection of the input impedances associated with the two descendent arteries. If an arterial segment is connected to a single descendent artery, its load impedance is given simply by the input impedance associated with the descendent artery. If an arterial segment itself is a terminal artery connected to a peripheral load, its load impedance is given by the impedance associated with the load. Full details of the TL model is provided in He et al. (2012). This model was validated with physiological data and the results of other studies, and was used in the study of arterial stenosis and arterial viscoelasticity (Xiao et al., 2016a, b, 2017).

### Creation of Virtual PAD Patients

**FIGURE 2:**
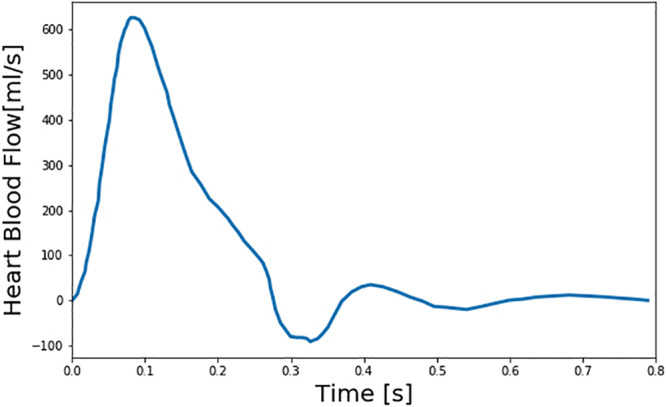
Representative heart blood flow waveform used as input to the multi-branch transmission line (TL) model of arterial hemodynamics associated with virtual patients.

We created a large number of virtual patients to investigate the potential and challenges in DL-based PWA for PAD diagnosis using the aforementioned multi-branch TL model. To create realistic virtual patients, we considered three layers of variabilities: inter-individual, intra-individual, and PAD severity. First, we considered the inter-individual variability in the arterial hemodynamics associated with the virtual patients by widely varying five anatomical and physiological parameters in the multi-branch TL model: arterial length, diameter, and thickness, arterial elasticity, and peripheral load resistance. These parameters were varied up to ±20% around the nominal values reported in He et al. (2012) in an increment of 10%, which resulted in a total of 5^5^ = 3125 virtual patients associated with 5^5^ distinct arterial hemodynamic properties. Second, we considered the PAD severity variability in each virtual patent by widely varying the degree of the artery occlusion in the multi-branch TL model. In this exploratory work, we limited our focus to PAD occurring in the abdominal aorta, which is one of the most common PAD sites. In each of the 3125 virtual patients, we included PAD by varying diameter associated with the abdominal aorta. We considered PAD severity of 0–80% in an increment of 10% for training and validation datasets and in an increment of 1% for test dataset, where severity is measured as the degree of artery area occlusion (0% implies no occlusion while 100% implies complete occlusion). This resulted in a total of 3125 × 9 = 28,125 virtual patients, associated with distinct arterial hemodynamics and PAD, as the basis to construct training and validation datasets and 3125 × 81 = 253,125 virtual patients, associated with distinct arterial hemodynamics and PAD, as the basis to construct test dataset. Third, we considered the intra-individual variability in the arterial hemodynamics in each virtual patient to account for the uncertainty due to model imperfection as well as random anatomical and physiological variations. We assumed that the five anatomical and physiological parameters in the multi-branch TL model used to account for the inter-individual arterial hemodynamic variability have log-normal distributions around the individual-specific values as mean values with coefficient of variation of 0.01 in each virtual patient. Finally, we constructed training and validation datasets by sampling 100 and 10 times from each of the 28,125 virtual patients equipped with random anatomical and physiological variations, and likewise constructed test dataset by sampling 10 times from each of the 253,125 virtual patients equipped with random anatomical and physiological variations. Then, we created arterial BP and BF waveforms associated with each of these samples by inputting a representative heart blood flow waveform used in He et al. (2012; Figure 2) to the multi-branch TL model characterized by the sample-specific anatomical and physiological parameters (including PAD severity). In this way, training and validation datasets were composed of 2,812,500 and 281,250 arterial BP and BF waveform data samples corresponding to 28,125 virtual patients, while test dataset was composed of 2,531,250 arterial BP and BF waveform data samples corresponding to 253,125 virtual patients.

### PAD Diagnosis via Deep Learning-Based Pulse Waveform Analysis

We developed our DL-based PWA approach to PAD diagnosis using the training and validation datasets constructed in section “Creation of Virtual PAD Patients.” Specifically, we constructed a deep CNN that can predict PAD severity by the analysis of arterial pulse waveforms. We in particular selected brachial and ankle BP waveforms as inputs to our deep CNN in order to make our approach compatible to the state-of-the-art ABI technique, so that (i) our approach and ABI can be directly compared and (ii) the potential for real-world application of our approach is maximized. Details follow.

**FIGURE 3:**
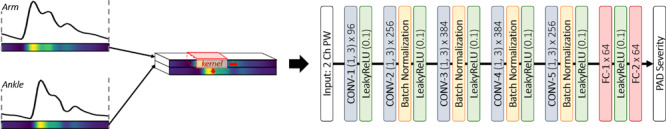
Deep convolutional neural network (CNN) architecture for PAD diagnosis via deep learning-based arterial pulse waveform analysis. CONV-*n* (*h*, *l*) × *k*: *n*th convolution layer with height *h*, length *l* and the number of kernel *k*. LeakyReLU (*a*): LeakyReLU activation with slope *a* on negative inputs. FC-*n* × *m*: *n*th fully connected layer with the number of node *m*.

Our deep CNN was built upon the AlexNet (Krizhevsky et al., 2012; Han et al., 2017; Wang et al., 2019), which was regarded as appropriate in dealing with 1-D arterial pulse waveforms associated with less complexity than 2-D images relative to other deeper CNN architectures such as ResNet (He et al., 2016) and DenseNet (Huang et al., 2017). To obviate extensive tuning of hyper-parameters, we adopted the original AlexNet architecture (five convolution layers and three fully connected layers), but with modest modifications (Figure 3). First, we employed the LeakyReLU as the activation function for the entire network to promote stable convergence in the training phase (Goodfellow et al., 2016). Second, we employed batch normalization in all the convolution layers to promote stable back propagation of gradient as well as regularization (Goodfellow et al., 2016). Third, we reduced the size of the fully connected layer to 64 to match it to the number of latent features outputted by the last convolution layer in our CNN. Using the network architecture thus specified, we constructed the deep CNN in such a way that brachial and ankle arterial pulses are convoluted independently (Figure 3). For this purpose, brachial and ankle arterial pulses undergo channel-wise concatenation so that these arterial pulses can be convoluted separately from each other by a shared kernel in the convolution layer. In this way, discriminative features of PAD severity embedded in the brachial and ankle arterial pulses can be extracted independently while computational efficiency can be gained with the use of shared kernels. In addition, mutual interactions between the discriminative features associated with the two arterial pulses can be exploited in the fully connected layer of the network.

To train the deep CNN, we used NVIDIA Titan Xp GPU and PyTorch libraries. We used the mean squared error loss between the true vs. model-predicted PAD severity as the cost function. We used the ADAM optimization (α = 0.9, β = 0.999) with initial learning rate of 0.0002. To assess the robustness of the deep CNN, we examined the sensitivity of the cost function with respect to the local perturbations in the hyper-parameters including the number (increased by 1.5 and 2 times) and size (increased by 1 and 2) of kernels in the convolution layer. Note that the deep CNN thus trained with the above regression cost can be used to both detect and assess the severity of PAD. In particular, it can be used to detect PAD simply by labeling PAD in terms of PAD severity (i.e., classifying a subject as PAD patient if the subject’s PAD severity exceeds a pre-specified PAD severity threshold).

### Evaluation

We evaluated our DL-based PWA approach to PAD diagnosis and compared its efficacy with the state-of-the-art ABI technique, in terms of PAD detection and severity assessment efficacy, using the test dataset constructed in section “Creation of Virtual PAD Patients.” Details follow.

First, we evaluated our approach for its PAD detection performance. We considered a range of PAD severity threshold levels in labeling healthy subjects and PAD patients (10–70%, in an increment of 10%). For each PAD labeling threshold level, we randomly selected 2000 virtual patients from test dataset (consisting of 253,125 virtual patients; see section “Creation of Virtual PAD Patients”) so that the selected patients include equal number of healthy subjects and PAD patients (i.e., 1000 healthy subjects and 1000 PAD patients; for example, in case of 40% PAD severity threshold for labeling, 1000 virtual patients with <40% PAD severity were randomly chosen to form healthy subjects while 1000 virtual patients with ≥40% PAD severity were randomly chosen to form PAD patients). Then, we evaluated our approach and ABI technique using the 20,000 arterial BP and BF waveform data of these 2000 virtual patients (see section “Creation of Virtual PAD Patients”) by (i) classifying each arterial BP and BF waveform data sample into healthy or PAD category based on the PAD severity predicted by the deep CNN when the brachial and ankle BP waveforms in the sample were inputted and the ABI value computed from the waveforms, (ii) aggregating the classification results across all the 20,000 data samples associated with all the 2000 virtual patients, and (iii) computing the sensitivity and specificity as well as the accuracy of PAD detection. In the context of PAD detection, sensitivity was defined as the proportion of the 10,000 PAD patient samples which were actually detected as such (with the PAD severity predicted to be higher than the PAD labeling threshold), while specificity was defined as the proportion of the 10,000 healthy subject samples which were actually detected as such (with the PAD severity predicted to be lower than the PAD labeling threshold). Accuracy was defined as the proportion of the 20,000 test samples whose labels were classified correctly.

Second, we evaluated our approach for its PAD severity assessment performance. We randomly selected 2,000 virtual patients from test dataset (consisting of 253,125 virtual patients; see section “Creation of Virtual PAD Patients”) so that the selected patients are distributed uniformly across all the PAD severity levels (1–80% in an increment of 1%, which amounts to 25 virtual patients per PAD severity level). Then, we evaluated our approach and ABI technique using the 20,000 arterial BP and BF waveform data samples of these 2000 virtual patients (see section “Creation of Virtual PAD Patients”), in terms of the Bland-Altman statistics between the true PAD severity vs. the PAD severity predicted by our deep CNN and ABI. To map ABI value to PAD severity, we pre-calibrated the ABI values to the corresponding PAD severity level based on a polynomial regression model relating ABI to PAD severity (which was obtained from the nominal virtual patient characterized by the nominal anatomical and physiological parameter values). Third, we analyzed the latent feature space associated with our deep CNN using the t-distributed stochastic neighbor embedding (t-SNE) algorithm. This analysis was conducted to examine the presence of a smooth manifold relating the latent features to PAD severity. We applied t-SNE to visualize the input space and the space of latent features at the last convolution layer into 2-dimensional space. Then, we investigated the distributions of the input and latent features in the 2-dimensional space for a connected manifold in the direction of PAD severity. Fourth, we analyzed our deep CNN using the gradient-weighted class activation mapping (GradCAM) algorithm (Selvaraju et al., 2017) to interpret the discriminative input features exploited by our deep CNN in predicting PAD severity. We applied GradCAM to visualize the discriminative features (i.e., regions) in the brachial and ankle arterial BP waveforms which largely contributed in predicting PAD severity. Then, we assessed the physiological relevance of the input features exploited by the deep CNN in diagnosing PAD by comparing these discriminative features and the available clinical knowledge on the relationship between PAD severity and arterial pulse waveforms.

To derive a robust estimate of detection and diagnosis performance, we repeated the above evaluation 10 times and reported the average values of the sensitivity, specificity, and accuracy as well as the Bland-Altman statistics.

## Results

**FIGURE 4:**
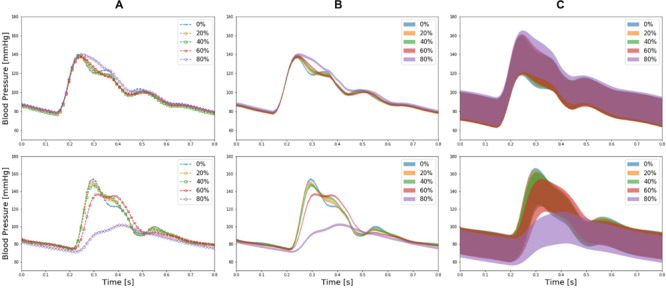
Brachial (upper panel) and ankle (lower panel) blood pressure (BP) waveforms corresponding to **(A)** nominal virtual patient (i.e., virtual patient with nominal anatomical and physiological parameter values), **(B)** nominal virtual patient with intra-individual variability, and **(C)** all the virtual patients with inter- and intra-individual variability in the test dataset, all associated with varying PAD severity levels.

**FIGURE 5:**
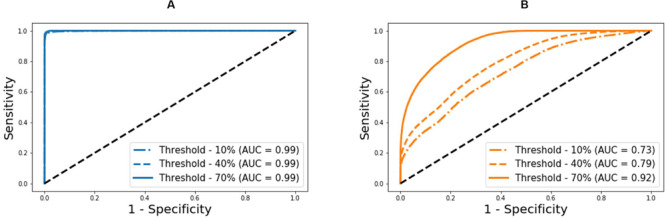
Receiver operating characteristic curves associated with the deep learning-based pulse waveform analysis approach and ankle-brachial index (ABI), both corresponding to varying PAD severity threshold levels for labeling of healthy subjects and PAD patients. **(A)** DL-based pulse waveform analysis approach. **(B)** Ankle-brachial index.

**FIGURE 6:**
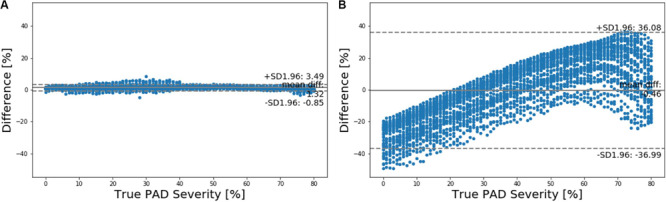
Bland-Altman plots between true PAD severity vs. PAD severity predicted by **(A)** deep learning-based pulse waveform analysis approach and **(B)** ankle-brachial index (ABI).

**FIGURE 7:**
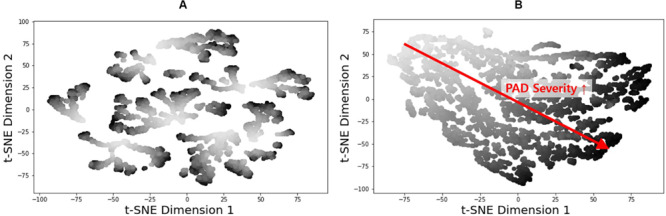
2-dimensional t-distributed stochastic neighbor embedding (t-NSE) visualization of **(A)** input and **(B)** latent feature spaces associated with the fully trained and validated deep convolutional neural network.

**FIGURE 8:**
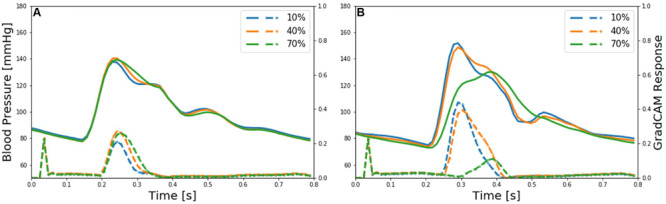
Representative brachial and ankle pulse waveforms (solid lines) and discriminative features (dotted lines) of deep convolutional neural network (CNN) localized by the gradient-weighted class activation mapping (GradCAM) associated with low (10%), medium (40%), and high (70%) PAD severity levels. **(A)** Brachial arterial pulse. **(B)** Ankle arterial pulse.

Figure 4 presents brachial and ankle BP waveforms corresponding to (a) nominal virtual patient, (b) nominal virtual patient with intra-individual variability, and (c) all the virtual patients with inter- and intra-individual variability in the test dataset, all associated with varying PAD severity levels. Table 1 summarizes the PAD detection performance of our approach and ABI (measured in terms of detection sensitivity, specificity, and accuracy), both corresponding to varying PAD severity threshold levels for labeling of healthy subjects and PAD patients. Figure 5 shows the receiver operating characteristic (ROC) curves associated with our approach and ABI, both corresponding to varying PAD severity threshold levels for labeling of healthy subjects and PAD patients. Figure 6 shows the Bland-Altman plots between true PAD severity vs. PAD severity predicted by our approach and ABI. Figure 7 presents the 2-dimensional t-SNE visualization of the input and latent feature spaces associated with the fully trained and validated deep CNN, while Figure 8 presents discriminative input features of our deep CNN localized by GradCAM associated with low and high PAD severity levels.

**TABLE 1:** PAD detection performance of the deep learning-based pulse waveform analysis approach and ankle-brachial index, both corresponding to varying PAD severity threshold levels for labeling of healthy subjects and PAD patients.

**Labeling threshold**		**10%**	**20%**	**30%**	**40%**	**50%**	**60%**	**70%**
DL	Sensitivity	0.97	0.96	0.94	0.95	0.93	0.92	0.85
	Specificity	0.99	0.99	0.99	0.99	0.99	0.99	0.99
	Accuracy	0.99	0.98	0.97	0.97	0.96	0.95	0.91
	AUC	0.99	0.99	0.99	0.99	0.99	0.99	0.99
ABI	Sensitivity	0.96	0.94	0.73	0.64	0.60	0.58	0.59
	Specificity	0.50	0.50	0.64	0.75	0.91	0.99	0.99
	Accuracy	0.50	0.51	0.68	0.68	0.66	0.64	0.65
	AUC	0.73	0.74	0.76	0.79	0.83	0.88	0.92

**DL, deep learning-based pulse waveform analysis approach; ABI, ankle-brachial index.**

## Discussion

PAD is a highly prevalent CVD with profound morbidity and mortality implications, but it is frequently undiagnosed due to the limitations associated with the cost, comfort, and accuracy of existing angiography and ABI techniques. In this work, we investigated an affordable, convenient, and accurate PAD screening and diagnosis approach via DL-based PWA. Using a large number of virtual patients created with a validated multi-branch TL model of arterial hemodynamics, we illustrated its potential and challenges to overcome.

### Validity of Virtual Patients

The virtual patients created with the multi-branch TL model of arterial hemodynamics could reproduce the clinically observed trends in the shape of the arterial pulse waveforms in response to varying degree of PAD severity. In particular, the multi-branch TL model predicted that ankle BP pulse undergoes the following morphological changes with an increase in the PAD severity level: (i) systolic peak flattens; (ii) secondary diastolic peak disappears; (iii) pulse amplitude decreases; (iv) crest time (time interval between diastolic trough and systolic peak) increases; and (v) pulse width at half amplitude increases (Figure 4). It also predicted that brachial pulse amplitude increases, which contributes to a decrease in ABI with an increase in the PAD severity level. These predictions are consistent with a number of existing clinical observations (Carter, 1968; Davies et al., 2014; Sumpio and Benitez, 2015; Dhanoa et al., 2016; Mao et al., 2017; Sibley et al., 2017) at least from qualitative standpoint. In sum, it was concluded that the virtual patients used in our work can produce realistically plausible arterial pulse waveforms with respect to varying degree of PAD severity, which provided a solid basis to investigate the strengths and weaknesses of our DL-based PWA approach to PAD screening and diagnosis especially in comparison with the widely used ABI technique.

### PAD Detection and Severity Assessment Efficacy

Our approach boasted robust PAD detection performance superior to the ABI technique against a wide range of PAD severity threshold levels for labeling of healthy subjects and PAD patients (Table 1 and Figure 5). The sensitivity, specificity, and accuracy values computed at the PAD classification threshold levels identical to the labeling threshold values [note that (i) the deep CNN was calibrated to the true PAD severity as part of training, and (ii) a PAD severity level can be mapped to its corresponding ABI by using the polynomial regression model relating ABI to PAD severity in section “Evaluation”] were consistently higher in our approach than the ABI technique (Table 1). Our approach also boasted PAD severity assessment performance largely superior to the ABI technique, as indicated by its much smaller limits of agreement between the true vs. predicted PAD severity levels in comparison to its ABI counterparts (Figure 6). Overall, it appears that ABI is susceptible to the inter-individual variability in anatomical and physiological parameters which affect the systolic peak values associated with brachial and ankle arterial pulses, whereas our approach can cope with those confounding factors via highly sophisticated analysis of the two arterial pulse waveforms to exploit morphological characteristics beyond systolic peak values. The PAD detection and severity assessment performance remained consistent against repeated tests: the sensitivity, specificity, and accuracy values exhibited small coefficients of variation of the order of 10^–3^ across the 10 repeated tests outlined in section “Evaluation.” Lastly, the deep CNN appeared to be robust against modest perturbations in its hyper-parameters in that the alteration in the cost function with respect to the hyper-parameter perturbations considered in this work was small (<2.3%). This suggests that the AlexNet architecture used in this work was adequate, if not ideal.

Our approach exhibited a tendency for slight underestimation of PAD severity, especially at high PAD severity levels (Figure 6A). This may explain its imperfect sensitivity relative to specificity at high PAD labeling threshold (Table 1), because underestimation of PAD severity in general makes the deep CNN conservative in detecting PAD. In contrast, the ABI technique suffered from a tendency for severe overestimation of PAD severity in low-severity PAD and also severe underestimation of PAD severity in high-severity PAD (Figure 6B). This may explain its deteriorating sensitivity and improving specificity (and the suboptimal accuracy as a whole) with respect to the increase in the PAD labeling threshold (Table 1). In our virtual patients, ABI tended to remain at a normal constant level up to ∼50% PAD severity level, beyond which it started to sharply decrease (not shown). Hence, the sensitivity of ABI is high in low PAD labeling thresholds (since it overestimates the severity in low PAD severity regime) but is low in high PAD labeling thresholds (since it underestimates the severity in high PAD severity regime). For the same reason, the specificity of ABI is low in low PAD labeling thresholds but is high in high PAD labeling thresholds. It is worth noting that this trend is in accordance with prior clinical observations on the low sensitivity and high specificity of ABI in detecting symptomatic PAD patients (Stein et al., 2006; Wikström et al., 2008).

### Latent Feature and Interpretability Analysis

Two inherent challenges associated with DL is its susceptibility to overfitting and lack of transparency. We employed (i) t-SNE to examine if our deep CNN was properly trained and (ii) GradCAM to examine if our deep CNN exploits appropriate input features in diagnosing PAD.

The t-SNE visualization of the input and latent feature spaces clearly illustrates that the deep CNN was properly trained to capture the relationship between the latent features extracted from the brachial and ankle pulse waveforms and PAD severity (Figure 7). In particular, the input feature space contains a number of small and scattered clusters associated with varying PAD severity levels (Figure 7A), which presumably represent the inter-individual variability associated with the virtual patients. In contrast, the latent feature space clearly shows a manifold smoothly connecting low (upper left) to high (lower right) PAD severity levels (Figure 7B). Hence, it may be claimed that the notable performance of the DL-based PWA approach originates from its appropriate learning of the latent features indicative of PAD severity rather than from overfitting to the data.

The discriminative input features localized by GradCAM provide support for the transparency of the deep CNN constructed in this work. Indeed, main discriminative input features included (i) the systolic up-stroke and (ii) diastolic down-stroke (including secondary peaks when exists) (Figure 8), which are the regions in the brachial and ankle arterial pulses in which salient morphological changes occur as PAD develops according to the existing clinical literature (Carter, 1968; Davies et al., 2014; Sumpio and Benitez, 2015; Dhanoa et al., 2016; Mao et al., 2017; Sibley et al., 2017). Hence, it can be claimed that the DL-based PWA approach may detect and assess the severity of PAD by analyzing brachial and ankle arterial pulse waveforms in a way similar to how experienced clinicians analyze them, although the exact mechanisms underlying how the deep CNN compiles and interprets the observed morphological changes into PAD severity are unknown.

### Limitations and Opportunities

All in all, this work demonstrated the proof-of-concept of integrating DL and PWA for affordable and non-invasive PAD screening and diagnosis. However, this work has a number of limitations to be addressed. In addition, this work also sheds light on outstanding opportunities toward its real clinical application.

First and foremost, this work was conducted using data collected from virtual rather than real patients. We employed a validated multi-branch TL model to create virtual patients. We also showed that arterial pulse waveforms produced by the virtual patients exhibit the morphological characteristics observed in real PAD patients. Yet, discrepancy between virtual vs. real patients may be inevitable at least to some extent, and there are a few potential sources that can obscure the initial success of this work when applied to real clinical data. In particular, the inter- and intra-individual variability considered in this work is somewhat ad-hoc. Furthermore, we accounted for variability associated only with arterial anatomical and physiological parameters but not cardiac parameters (such as stroke volume and ejection duration). In the near term, the efficacy of our approach against variabilities not considered in this work may be investigated using the same virtual patients. But ultimately, future work must confirm the proof-of-concept obtained in this work using clinical data collected from real patients. Regardless of this limitation, this work may still have unique value as an exploratory study of DL-based arterial pulse waveform analysis for PAD diagnosis in a reasonably realistic yet resource-effective and controlled setting. Indeed, our work may provide a strong justification for conducting a (potentially large-scale and resource-intensive) clinical data collection study for experimental investigation of DL-based PWA approaches to PAD diagnosis (and perhaps other CVDs as well).

Second, this work was limited to the detection and severity assessment of PAD in a single arterial site. In contrast, an ideal PAD screening and diagnosis tool is required to also localize PAD. Hence, our approach must be extended to a technique capable of simultaneously detecting, localizing, and assessing the severity of PAD. This requirement may present additional challenge when PAD at multiple sites with different levels of severity must be diagnosed. Future work must investigate how to extend our approach to also include PAD localization capability. A possible initial strategy may be to leverage the deep CNN trained in this work in conjunction with the multi-task learning, pre-training, and continuation methods established in the DL domain so as to extend the current deep CNN to also embed the ability to localize PAD.

Third, this work assumed the availability of a large amount of data associated with a wide range of variability in anatomical and physiological characteristics as well as PAD severity levels, which may not be practically realistic. For example, the majority of PAD data may be associated with aged patients, and our approach when trained with such data may not generalize well to young patients (who are associated with low PAD incidence but screening/diagnosing whom is still crucial for CV risk management). Likewise, our approach when trained with data associated with one ethnic population may not generalize well to another subject to a large inter-ethnic anatomical and physiological discrepancies. Future work on coping with limited data and enormous inter-individual variability must be conducted. A possible initial strategy may be to exploit the domain adaptation and transfer techniques as well as adversarial training to guide the deep CNN work with latent features invariant to ethnic, anatomical, and physiological characteristics.

Lastly, this work used arterial BP waveforms, which may not be easy to measure non-invasively. Practically affordable non-invasive arterial pulse waveforms (e.g., pulse volume recording waveforms; Davies et al., 2014; Sumpio and Benitez, 2015; Ghasemi et al., 2018) are typically measured at the skin level and thus exhibit subtle morphological differences relative to arterial BP waveforms (Lee et al., 2018). Hence, future work must be conducted to investigate adverse effect of using non-invasive arterial pulse waveform measurements on our approach as well as innovative strategies to realize our approach using affordable and non-invasive arterial pulse measurements.

## Conclusion

This work demonstrated the proof-of-concept of a novel DL-based PWA approach to PAD diagnosis. The results suggest that PAD detection and severity assessment may be feasible with data-driven analysis of arterial pulse waveforms. This work also outlined outstanding opportunities and challenges toward real-world deployment of our approach, including (i) validation with data collected from real patients, (ii) PAD localization, (iii) generalizable implementation with limited data and robustness against confounding factors, and (iv) practical embodiment with affordable and non-invasive arterial pulse waveforms. Future work to explore and address these opportunities and challenges, including the development of innovative DL-based PWA algorithms capable of addressing the outstanding obstacles, may serve as key cornerstones to realize affordable and convenient PAD screening and diagnosis.

## Data Availability Statement

The datasets generated for this study are available on request to the corresponding author.

## Author Contributions

SK, J-OH, and BY conceived the study and analyzed and interpreted the results. SK and J-OH created the virtual PAD patient data, developed the DL-based pulse wave analysis, and wrote and revised the manuscript. BY reviewed the manuscript. All authors contributed to the article and approved the submitted version.

## Conflict of Interest

BY was employed by company OnePredict, Inc. The remaining authors declare that the research was conducted in the absence of any commercial or financial relationships that could be construed as a potential conflict of interest.
